# Supplementary material for: The Evolution and Characterization of the RNA Interference Pathways in Lophotrochozoa
Source: Genome Biol Evol. 2024 May 7;16(5):evae098. doi: 10.1093/gbe/evae098 (PMC11114477; doi:10.1093/gbe/evae098)
Supplement: evae098_Supplementary_Data [file evae098_supplementary_data.zip › Supplementary_materials.zip!/selected_miRNAs/NoGe_selected_miRNAs/mirdeep_pdf_files/NMRB01000012.1_Notospermus_geniculatus_strain_Ushimado_scaffold12,_whole_genome_shotgun_sequence_567.pdf]

Provisional ID	:	NMRB01000012.1_Notospermus_geniculatus_strain_Ushimado_scaffold12,_whole_genome_shotgun_sequence_567
Score total	:	5344.4
Score for star read(s)	:	3.9
Score for read counts	:	5336.5
Score for mfe	:	1.1
Score for randfold	:	0
Score for cons. seed	:	3
Total read count	:	10479
Mature read count	:	10398
Loop read count	:	0
Star read count	:	81

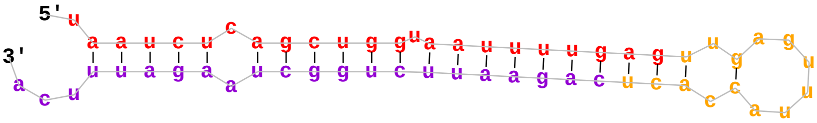

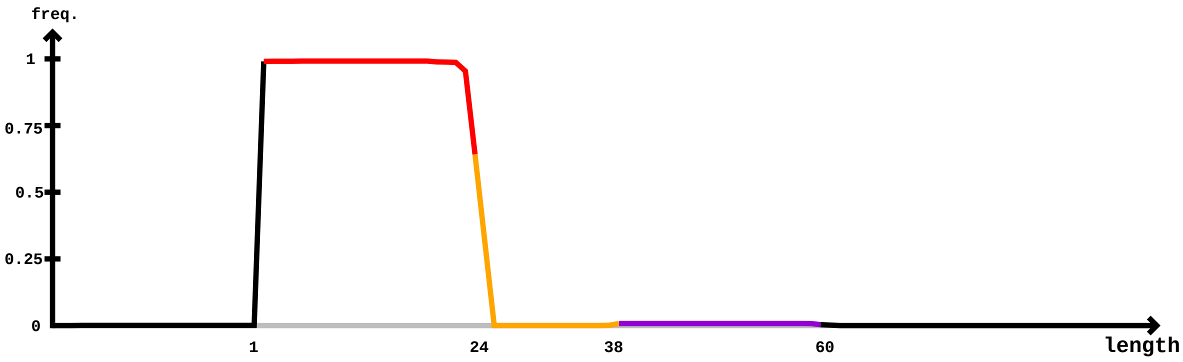

Mature Star

```
5'- cuaauuguuggacguuugucuaaucucagcugguaauuuugaguugaguuuaccacucagaauucuggcuaagauuucaugaaugucuauauuaacccuuuagcaugcuguua  -3'   obs
    cuaauuguuggacguuugucuaaucucagcugguaauuuugaguugaguuuaccacucagaauucuggcuaagauuucaugaaugucuauauuaacccuuuagcaugcuguua         exp
    ........((((((((((..(((((.((((((.((((((((((.(......).))))))))))))))).))))).))))))))))..........(((((...)))))    reads   mm   sample
    .uaauuguuggacguuuguc............................................................................................. 1       0    Fm3
    ....................uaaucucagcugguaauu........................................................................... 13      0    Fm3
    ....................uaaucucagcugguaauuu.......................................................................... 4       0    Fm3
    ....................uaaucucagcugguaauuuu......................................................................... 2       0    Fm3
    ....................uaaucucagcugguaauuuug........................................................................ 39      0    Fm3
    ....................uaaucucagcugguaaCuuug........................................................................ 1       1    Fm3
    ....................uaaucucagcugguaaGuuug........................................................................ 1       1    Fm3
    ....................uaaucucagcugguaauAuuga....................................................................... 2       1    Fm3
    ....................uaaucucagcugguGauuuuga....................................................................... 1       1    Fm3
    ....................uaaucuAagcugguaauuuuga....................................................................... 1       1    Fm3
    ....................uaaucucagcugguaauuuuga....................................................................... 240     0    Fm3
    ....................uaaucucagcugguaauuCuga....................................................................... 17      1    Fm3
    ....................uaaucucagcuUguaauuuuga....................................................................... 1       1    Fm3
    ....................uaaucucagcugguaauGuuga....................................................................... 2       1    Fm3
    ....................Naaucucagcugguaauuuuga....................................................................... 1       1    Fm3
    ....................uaaucucagcugguaaCuuuga....................................................................... 1       1    Fm3
    ....................uaaucucagcGgguaauuuuga....................................................................... 1       1    Fm3
    ....................uaaucucagcugguaauuuGga....................................................................... 21      1    Fm3
    ....................uaaucucagcugguaaAuuuga....................................................................... 1       1    Fm3
    ....................uaaucucagcugguaauuuugaA...................................................................... 7       1    Fm3
    ....................uaaucucagcugguaauuuugag...................................................................... 192     0    Fm3
    ....................uaaucucagcugguaauGuugag...................................................................... 4       1    Fm3
    ....................uaaucucagcugguaauCuugag...................................................................... 1       1    Fm3
    ....................uaaucucagcugguaauuuugagA..................................................................... 1       1    Fm3
    ....................uaaucucagcugguaaGuuugagu..................................................................... 1       1    Fm3
    ....................uaaucucagcugguaauuuugagu..................................................................... 7       0    Fm3
    ....................uaaucucagcugguaauuuugaAu..................................................................... 1       1    Fm3
    ........................cucagcugguaauuCuga....................................................................... 7       1    Fm3
    .........................................................cagaauucuggcuaagauuuca.................................. 1       0    Fm3

    .uaauuguuggacguuuguc............................................................................................. 4       0    Fm1
    ....................uaaucucagcugguaauu........................................................................... 3       0    Fm1
    ....................uaaucucagcugguaauuu.......................................................................... 3       0    Fm1
```

Mature Star

cuaauuguuggacguuugucuaaucucagcugguaauuuugaguugaguuuaccacucagaauucuggcuaagauuucaugaaugucuauauuaacccuuuagcaugcuguua			
....................uaaucucagcGgguaauuuug..	1	1	Fm1
....................uaaucucagcugguaauuuug..	39	0	Fm1
....................uaaucucagcugguaauuuGg..	2	1	Fm1
....................uaaucucagcugguaaAuuuga...	1	1	Fm1
....................uaaucucagcugguaauGuuga...	2	1	Fm1
....................uaaucucaCcugguaauuuuga...	1	1	Fm1
....................uaaucucagcugguaaCuuuga...	2	1	Fm1
....................uaaucucagcugguaauuuuga...	470	0	Fm1
....................uaaucucagcugguaauuCuga...	1	1	Fm1
....................uaaucucagcAgguaauuuuga...	1	1	Fm1
....................uaaucucagcugguaauuuGga...	4	1	Fm1
....................uaaucucagcugguaauuuugG...	2	1	Fm1
....................uaaucucagcugguaauCuuga...	2	1	Fm1
....................uaaucucagcugguaauAuuga...	3	1	Fm1
....................uaaCcucagcugguaauuuuga...	1	1	Fm1
....................uaaucucagcugguaauuuugU...	6	1	Fm1
....................uaaucucagcGgguaauuuugag..	2	1	Fm1
....................uaaucucagcugguGauuuugag..	1	1	Fm1
....................uaaucucagcugguUauuuugag..	1	1	Fm1
....................uaaucAcagcugguaauuuugag..	1	1	Fm1
....................uaaucucagcugguaGuuuugag..	1	1	Fm1
....................uaaucucagcugguaauCuugag..	12	1	Fm1
....................uaaucucagcugguaaGuuugag..	4	1	Fm1
....................Naaucucagcugguaauuuugag..	1	1	Fm1
....................uaaucNcagcugguaauuuugag..	1	1	Fm1
....................uaaucucagAugguaauuuugag..	1	1	Fm1
....................uaaucucagcAgguaauuuugag..	2	1	Fm1
....................uaaucucagcugguaauuuugUg..	1	1	Fm1
....................uaaucucagcugguaaAuuugag..	5	1	Fm1
....................uaaCcucagcugguaauuuugag..	1	1	Fm1
....................uaaucucagcugguaauuuugag..	1784	0	Fm1
....................uaaucucagcuggAaauuuugag..	1	1	Fm1
....................uaaucucagcCgguaauuuugag..	1	1	Fm1
....................uaaucuGagcugguaauuuugag..	1	1	Fm1
....................uaCucucagcugguaauuuugag..	1	1	Fm1
....................uUaucucagcugguaauuuugag..	2	1	Fm1
....................uaaucuUagcugguaauuuugag..	5	1	Fm1
....................uaaucucagcugguaaCuuugag..	8	1	Fm1
....................uaaucucagcugguaauGuugag..	8	1	Fm1
....................uaaucucagcugguaauAuugag..	10	1	Fm1
....................uaaucucagcugguaauuuugaA..	14	1	Fm1
....................uaaucucGgcugguaauuuugag..	1	1	Fm1
....................uaaucucaUcugguaauuuugag..	1	1	Fm1
....................uaaucucagcugguaauuuugaU..	3	1	Fm1
....................uaaucucagcugguaauuuugagu...	65	0	Fm1
....................uaaucucagcugguaauCuugagu...	2	1	Fm1
....................uaaucucagcugguaauuuugagA...	6	1	Fm1
....................uaaucucagcugguaauuuugaAu...	2	1	Fm1
....................uaaucucagcugguaaCuuugagu...	2	1	Fm1
....................uaaucucagcugguaauuuugagC...	4	1	Fm1
.....................aaucucagcugguaauuuugag..	1	0	Fm1
....ucagaauucuggcuaagauuuc...................................	2	0	Fm1
....cagaauucuggcuaagauuuc...................................	10	0	Fm1
....cagaauucuggcuGagauuuca..................................	2	1	Fm1
....cagaauucuggcuaagauuuca..................................	2	0	Fm1
....................uaaucucagcugguaauu...	9	0	Fm2
....................uaaucucagcugguaauuu..	2	0	Fm2
....................uaaucucagcugguaauuCug..	3	1	Fm2
....................uaaucucagcugguaauuuuU..	1	1	Fm2
....................uaaucucagcugguaauCuug..	2	1	Fm2
....................uaaucucagcugguaauuAug..	1	1	Fm2
....................uaaucucagcugguaauuuug..	154	0	Fm2
....................uaaucucagcugguaauGuug..	1	1	Fm2
....................uaaucucagcugguaauAuug..	1	1	Fm2
....................uaaucucagcugguaauuuGg..	1	1	Fm2
....................uaaucucagcuAguaauuuuga...	1	1	Fm2
....................uaaucucagcugguaaAuuuga...	2	1	Fm2
....................uaaucucagcugguaauuuugG...	17	1	Fm2
....................uaaucucagcGgguaauuuuga...	1	1	Fm2

```
                    Mature                                  Star
cuaauuguuggacguuugucuaaucucagcugguaauuuugaguugaguuuaccacucagaauucuggcuaagauuucaugaaugucuauauuaacccuuuagcaugcuguua
....................uaaucucagcugguaauGuuga.......................................................................  12    1  Fm2
....................uaGucucagcugguaauuuuga.......................................................................  1     1  Fm2
....................uaauUucagcugguaauuuuga.......................................................................  1     1  Fm2
....................uaaucucagcugguaauAuuga.......................................................................  6     1  Fm2
....................uaauAucagcugguaauuuuga.......................................................................  1     1  Fm2
....................uaaucucagcugguaauuuuga.......................................................................  1533  0  Fm2
....................uaaucucagcugguaauuuGga.......................................................................  10    1  Fm2
....................uaaucucagcugguaauCuuga.......................................................................  14    1  Fm2
....................uaaucucagcugguaauuCuga.......................................................................  7     1  Fm2
....................uaaucucagcCgguaauuuuga.......................................................................  1     1  Fm2
....................uaUucucagcugguaauuuuga.......................................................................  2     1  Fm2
....................uaaucucagcuUguaauuuuga.......................................................................  1     1  Fm2
....................uaCucucagcugguaauuuuga.......................................................................  1     1  Fm2
....................uaaucucagcugguaaGuuuga.......................................................................  7     1  Fm2
....................uaaucucagcAgguaauuuuga.......................................................................  1     1  Fm2
....................uGaucucagcugguaauuuuga.......................................................................  1     1  Fm2
....................uaaucucagUugguaauuuuga.......................................................................  1     1  Fm2
....................uaaucucagcugguaaCuuuga.......................................................................  12    1  Fm2
....................uaaucucagcAgguaauuuugag......................................................................  2     1  Fm2
....................uaaucCcagcugguaauuuugag......................................................................  2     1  Fm2
....................uaaucucagcugguaauuuuUag......................................................................  1     1  Fm2
....................uaaucucagcugguaaAuuugag......................................................................  4     1  Fm2
....................uaaucucagcGgguaauuuugag......................................................................  2     1  Fm2
....................uaaucucagcugguaaCuuugag......................................................................  5     1  Fm2
....................uaaucucagcugguaaGuuugag......................................................................  4     1  Fm2
....................uaaucucagcugguaUuuuugag......................................................................  1     1  Fm2
....................uUaucucagcugguaauuuugag......................................................................  3     1  Fm2
....................uaGucucagcugguaauuuugag......................................................................  1     1  Fm2
....................uaaucucagcugguaauAuugag......................................................................  22    1  Fm2
....................uaaucucagcugguaauGuugag......................................................................  15    1  Fm2
....................uaaucucagcugguaauuuugaA......................................................................  45    1  Fm2
....................uaaucucagcugguaauuuugag......................................................................  3237  0  Fm2
....................uaaucucagcugguaauCuugag......................................................................  24    1  Fm2
....................uaaucucagAugguaauuuugag......................................................................  1     1  Fm2
....................uaaucucagcCgguaauuuugag......................................................................  4     1  Fm2
....................uaaucucagcugguaauuuuAag......................................................................  1     1  Fm2
....................uGaucucagcugguaauuuugag......................................................................  1     1  Fm2
....................uaaucucagcugguaauuuugUg......................................................................  1     1  Fm2
....................uaauAucagcugguaauuuugag......................................................................  1     1  Fm2
....................uaaucuGgcugguaauuuugag......................................................................  1     1  Fm2
....................uaUucucagcugguaauuuugag......................................................................  1     1  Fm2
....................uaaucucagcuUguaauuuugag......................................................................  1     1  Fm2
....................Naaucucagcugguaauuuugag......................................................................  1     1  Fm2
....................uaaucucagUugguaauuuugag......................................................................  1     1  Fm2
....................uaaucucagcugguaauuuugGg......................................................................  3     1  Fm2
....................uaaucAcagcugguaauuuugag......................................................................  1     1  Fm2
....................uaaucucaUcugguaauuuugag......................................................................  2     1  Fm2
....................uaaucuUgcugguaauuuugag.......................................................................  1     1  Fm2
....................uaaucucagcuggCaauuuugag......................................................................  2     1  Fm2
....................uaaucucagcugguaauuuugaU......................................................................  1     1  Fm2
....................uaaucucagcGgguaauuuugagu.....................................................................  1     1  Fm2
....................uaaucucagcugguaauuuugagu.....................................................................  123   0  Fm2
.....................aaucucagcugguaauuuugag......................................................................  4     0  Fm2
........................................................cagaauucuggcuaagauuuc....................................  14    0  Fm2
........................................................cagaauucuggcuaagauuuca...................................  13    0  Fm2

....................uaaucucagcugguaauu...........................................................................  2     0  Mm3
....................uaaucucagcugguaauGuu.........................................................................  1     1  Mm3
....................uaaucucagcugguaauuuu.........................................................................  4     0  Mm3
....................uaaucucagcugguaauuuug........................................................................  44    0  Mm3
....................uaaucucagcCgguaauuuuga.......................................................................  3     1  Mm3
....................uaaucucagcugguaauuCuga.......................................................................  1     1  Mm3
....................uaaucucagcugguaauGuuga.......................................................................  5     1  Mm3
....................uaaucucagcugguaauCuuga.......................................................................  1     1  Mm3
....................uaaucucagcGgguaauuuuga.......................................................................  1     1  Mm3
....................uaaucucagcugguaauuuGga.......................................................................  2     1  Mm3
....................uaaucucagcugguaauuuugU.......................................................................  1     1  Mm3
....................uaaucucagcugguaauuuuga.......................................................................  372   0  Mm3
....................uaaucucagcugguaaAuuuga.......................................................................  1     1  Mm3
....................uaaucucagcugguaauuuugG.......................................................................  4     1  Mm3
```

```
                    Mature                Star
cuaauuguuggacguuugucuaaucucagcugguaauuuugaguugaguuuaccacucagaauucuggcuaagauuucaugaaugucuauauuaacccuuuagcaugcuguua
....................uaaucucagcugguaaGuuuga.......................................................................   1     1   Mm3
....................uaaucucagcugguaaCuuugag......................................................................   1     1   Mm3
....................uaaucucagcugguaaAuuugag......................................................................   1     1   Mm3
....................Naaucucagcugguaauuuugag......................................................................   1     1   Mm3
....................uaGucucagcugguaauuuugag......................................................................   1     1   Mm3
....................uaaucucagcugguaauuuugag......................................................................   301   0   Mm3
....................uaaucucagcugguaauuuugaU......................................................................   3     1   Mm3
....................uaaucucagcugguaauAuugag......................................................................   1     1   Mm3
....................uaaucucagcugguaauuuugaA......................................................................   16    1   Mm3
....................uaaucucagcugguaauuuugUg......................................................................   1     1   Mm3
....................uaaucucagcugguaauGuugag......................................................................   2     1   Mm3
....................uaaucucagcugguaauCuugag......................................................................   1     1   Mm3
....................uaaucucagcugguaauuuugagu.....................................................................   10    0   Mm3
....................uaaucucagcugguaauuuugaUu.....................................................................   1     1   Mm3
....................uaaucucagcugguaauuuugaAu.....................................................................   1     1   Mm3
.....................aaucucagcugguaauuuugagu.....................................................................   1     0   Mm3
........................................................ucagaauucuggcuaagauuuc...................................   7     0   Mm3
.........................................................cagaauucuggcuaagauuuc...................................   1     0   Mm3
.........................................................cagaauucuggcuaagauuuca..................................   7     0   Mm3
.........................................................Uagaauucuggcuaagauuuca..................................   1     1   Mm3

....................uaaucucagcugguaauu...........................................................................   1     0   Mm1
....................uaaucucagcugguaauA...........................................................................   1     1   Mm1
....................uaaucucagcugguaaGu...........................................................................   1     1   Mm1
....................uaaucucagcugguaauuuu.........................................................................   1     0   Mm1
....................uaaucucagcugguaauuuug........................................................................   11    0   Mm1
....................uaaucucagcugguaauuCuga.......................................................................   1     1   Mm1
....................uaaucucagcugguaaGuuuga.......................................................................   1     1   Mm1
....................uaaucucUgcugguaauuuuga.......................................................................   1     1   Mm1
....................uaaucucagcugguaauCuuga.......................................................................   1     1   Mm1
....................uaaucucagcugguaaAuuuga.......................................................................   1     1   Mm1
....................uaaucucagcugguaauAuuga.......................................................................   1     1   Mm1
....................uaaucucagcugguaauuuuga.......................................................................   123   0   Mm1
....................uaaucucagcugguaauuuugG.......................................................................   1     1   Mm1
....................uaaucucagcugguaauuuugag......................................................................   87    0   Mm1
....................uaaucuGagcugguaauuuugag......................................................................   1     1   Mm1
....................uaaucucagcugguaauuuugaA......................................................................   6     1   Mm1
....................uaaucucagcugguaauuuugagu.....................................................................   5     0   Mm1

....................uaaucucagcugguaauu...........................................................................   2     0   Mm2
....................uaaucucagcugguaauuu..........................................................................   2     0   Mm2
....................uaaucucagcugguaauuuu.........................................................................   1     0   Mm2
....................uaaucucagcugguaauuCug........................................................................   1     1   Mm2
....................uaaucucagcugguaauCuug........................................................................   1     1   Mm2
....................uaaucucagcugguaauuuGg........................................................................   1     1   Mm2
....................uaaucucagcugguaauuuug........................................................................   37    0   Mm2
....................uaaucucagcugguaauuuugG.......................................................................   5     1   Mm2
....................uaaucucagcugguaaCuuuga.......................................................................   1     1   Mm2
....................uaaucucGgcugguaauuuuga.......................................................................   1     1   Mm2
....................uaaucucagcugguaauuCuga.......................................................................   1     1   Mm2
....................uaaucucagcugAuaauuuuga.......................................................................   1     1   Mm2
....................uaaucucagcugguaauGuuga.......................................................................   1     1   Mm2
....................uaaucucagcugguaauuuuga.......................................................................   303   0   Mm2
....................uaaucucagcugguaaAuuuga.......................................................................   1     1   Mm2
....................uUaucucagcugguaauuuuga.......................................................................   1     1   Mm2
....................uaaucucagcugguaauCuuga.......................................................................   1     1   Mm2
....................uaaucucagcugguaauuuugU.......................................................................   1     1   Mm2
....................uaaucucagcugguaauuuGga.......................................................................   5     1   Mm2
....................uaaucucagcGgguaauuuuga.......................................................................   1     1   Mm2
....................uaaucucagcugguaauAuuga.......................................................................   3     1   Mm2
....................uaaucucGgcugguaauuuugag......................................................................   1     1   Mm2
....................uaaucucagcugguaauuuugaA......................................................................   9     1   Mm2
....................uaaucucagcugguaauuAugag......................................................................   1     1   Mm2
....................uaaucucagcugguaauGuugag......................................................................   3     1   Mm2
....................uaaucucagcugguaauAuugag......................................................................   3     1   Mm2
....................uaaucucagcugguaaAuuugag......................................................................   1     1   Mm2
....................uaaucucagcugguaauuuGgag......................................................................   1     1   Mm2
....................uaaucucagcGgguaauuuugag......................................................................   1     1   Mm2
....................uaaucucagcugguaauCuugag......................................................................   4     1   Mm2
....................uaaucucagcugguaaGuuugag......................................................................   3     1   Mm2
```

```
                    Mature                                  Star
cuaauuguuggacguuugucuaaucucagcugguaauuuugaguugaguuuaccacucagaauucuggcuaagauuucaugaaugucuauauuaacccuuuagcaugcuguua
....................uaaAcucagcugguaauuuugag......................................................................   1     1     Mm2
....................uaaucucagcAgguaauuuugag......................................................................   1     1     Mm2
....................uaaucucagcugguaauuuugag......................................................................   553   0     Mm2
....................uaaucCcagcugguaauuuugag......................................................................   1     1     Mm2
....................uaaucucagcugguaauuuugagA.....................................................................   3     1     Mm2
....................uaaucucagcugguaauuuugagu.....................................................................   12    0     Mm2
....................uaaucucagcugguaauuuugaguu....................................................................   1     0     Mm2
.....................aaucucagcugguaauuuugag......................................................................   1     0     Mm2
........................................................ucagaauucuggcuaagauuuc...................................   3     0     Mm2
.........................................................cagaauucuggcuaagauuu....................................   2     0     Mm2
.........................................................cagaauucuggcuaagauuuc...................................   6     0     Mm2
.........................................................cagaauucuggcuaagauuuca..................................   10    0     Mm2
```
